# Breast cancer screening with digital breast tomosynthesis: comparison of different reading strategies implementing artificial intelligence

**DOI:** 10.1007/s00330-022-09316-y

**Published:** 2022-12-11

**Authors:** Victor Dahlblom, Magnus Dustler, Anders Tingberg, Sophia Zackrisson

**Affiliations:** 1 Diagnostic Radiology, Department of Translational Medicine, Lund University, Carl-Bertil Laurells gata 9, 205 02 Malmö, Sweden; 2 Department of Medical Imaging and Physiology, Skåne University Hospital, Malmö, Sweden; 3 Medical Radiation Physics, Department of Translational Medicine, Lund University, Malmö, Sweden; 4 Radiation Physics, Skåne University Hospital, Malmö, Sweden

**Keywords:** Mammography, Breast neoplasms, Early detection of cancer, Artificial intelligence, Electronic data processing

## Abstract

**Objectives:**

Digital breast tomosynthesis (DBT) can detect more cancers than the current standard breast screening method, digital mammography (DM); however, it can substantially increase the reading workload and thus hinder implementation in screening. Artificial intelligence (AI) might be a solution. The aim of this study was to retrospectively test different ways of using AI in a screening workflow.

**Methods:**

An AI system was used to analyse 14,772 double-read single-view DBT examinations from a screening trial with paired DM double reading. Three scenarios were studied: if AI can identify normal cases that can be excluded from human reading; if AI can replace the second reader; if AI can replace both readers. The number of detected cancers and false positives was compared with DM or DBT double reading.

**Results:**

By excluding normal cases and only reading 50.5% (7460/14,772) of all examinations, 95% (121/127) of the DBT double reading detected cancers could be detected. Compared to DM screening, 27% (26/95) more cancers could be detected (*p* < 0.001) while keeping recall rates at the same level. With AI replacing the second reader, 95% (120/127) of the DBT double reading detected cancers could be detected—26% (25/95) more than DM screening (*p* < 0.001)—while increasing recall rates by 53%. AI alone with DBT has a sensitivity similar to DM double reading (*p* = 0.689).

**Conclusion:**

AI can open up possibilities for implementing DBT screening and detecting more cancers with the total reading workload unchanged. Considering the potential legal and psychological implications, replacing the second reader with AI would probably be most the feasible approach.

**Key Points:**

• *Breast cancer screening with digital breast tomosynthesis and artificial intelligence can detect more cancers than mammography screening without increasing screen-reading workload.*

• *Artificial intelligence can either exclude low-risk cases from double reading or replace the second reader.*

• *Retrospective study based on paired mammography and digital breast tomosynthesis screening data.*

## Introduction

Digital breast tomosynthesis (DBT) has been shown to have a higher sensitivity for breast cancer detection than the current standard two-view digital mammography (DM) with mediolateral oblique (MLO) and craniocaudal (CC) views [1–3]; however, because it is a more complex examination with multiple slices causing a longer reading time, DBT is a more resource-intensive process [4]. Double reading is practiced in many screening programmes, especially in Europe, further amplifying the workload [5]. Moreover, the radiation dose with DBT is generally higher than with DM [3]. The radiation dose of one-view wide-angle DBT (MLO) is lower than standard two-view DM but has a higher sensitivity and similar performance to two-view DBT combined with two-view DM [1]. One-view wide-angle DBT screening reduces the interval cancer rate, which is often used as a surrogate measure for breast cancer mortality [6]. Many studies also suggest the use of two-view DBT, which avoids the risk of any information only available in the CC-view being lost and can result in an even higher sensitivity, although it can have a higher radiation dose than DM [2, 3, 7, 8]. However, a slightly higher dose might be acceptable if it meant a gain in sensitivity. Apart from radiation dose, the major remaining obstacle to implementing full DBT screening is the increased workload caused by longer reading time. In previous studies, the reading time for two-view DBT was 38–76% longer than for DM [7–11].

The use of artificial intelligence (AI) for the interpretation of DM examinations has shown promising results, both as a decision support tool for a reading radiologist [12, 13] and as a stand-alone reader [14–17]. AI as a stand-alone reader has been proposed to identify normal cases (cases which could safely be excluded from radiologist readings [14, 15]), to replace the second reader [16, 17], and to identify high-risk cases for more thorough assessment [15, 16]. While there are several studies of AI for DM, studies of AI for DBT are relatively scarce, with only a few studies of DBT on screening material at the time of writing, including two focusing on DBT reading workload reduction [18–21]. Several reader studies on the use of AI as decision support for DBT with cancer-enriched datasets have shown a reduction in reading time per examination with maintained or increased accuracy [22–27]. An AI model for predicting future short-term cancer risk from DBT has also been developed [28].

If AI could ease the burden of reading DBT, this could open up possibilities for the broad introduction of DBT in population-based screening programmes. The overall aim of this study is to retrospectively assess the cancer detection performance of a commercially available AI system on a single-view, wide-angle DBT screening material with paired two-view DM screening as a reference. More specifically, we will investigate our hypotheses that AI can be used to make DBT screening more efficient in terms of reading workload, by identifying normal cases that can be excluded from human reading, replacing the second reader, or replacing both readers. This is compared to DM and DBT screening with double reading, and the characteristics of detected and missed cancers are studied.

## Methods

### Study population

This retrospective study used data from the prospective population-based screening trial Malmö Breast Tomosynthesis Screening Trial (MBTST; ClinicalTrials.gov number NCT01091545) [1]. A total of 14,848 women were examined with both one-view wide-angle DBT (MLO) and two-view DM (MLO + CC) with separate double reading and recall decisions after consensus discussion. The readers (seven radiologists with 2–41 years of experience in breast radiology) used a five-point cancer probability scale (1: no abnormalities, 2: benign findings, 3: non-specific findings with low cancer probability, 4: cancer-suspicious findings, 5: highly cancer-suspicious findings), later called the “radiologist score”. Consensus discussions were held for all cases with values ≥ 3 by at least one reader or if marked for discussion by at least one reader. Decisions from each reader were extracted from the radiology information system. Reading time data were unavailable. All examinations were performed with a Mammomat Inspiration (Siemens Healthineers). Cancers diagnosed during the follow-up until the next screening (18 or 24 months, depending on age) were included as interval cancers. The study was approved by the Local Ethics Committee at Lund University (official records number: 2009/770).

**Fig. 1:**
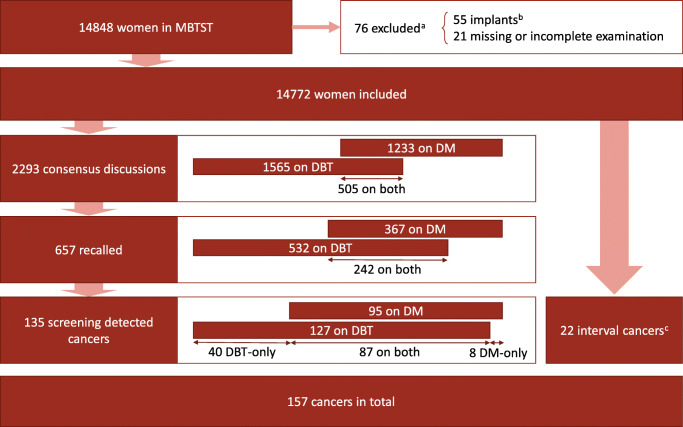
Chart of the study population, including exclusions, recalls, screening-detected cancers, and interval cancers. ^a^Including two cancers. ^b^Not supported by the AI system. ^c^Interval cancers detected during a follow-up period of 1.5 or 2 years, depending on age

Some examinations had to be excluded from the present study, as illustrated in more detail in Figure 1, together with the number of consensus discussions, recalls and screening-detected cancers for DM and DBT reading arms. In total, 14,772 women were included, with 157 women diagnosed with cancer, including 135 screening-detected cancers and 22 interval cancers.

### Artificial intelligence system

The DBT examinations were analysed separately from DM using the commercially available DM and DBT AI system Transpara v1.7.0 (ScreenPoint Medical) [12, 14, 19, 26, 27, 29]. The system classifies each examination with a score between 0 and 9.99. The system was not trained on any data from the studied population. In this study, the AI system was used retrospectively as a stand-alone reader. In clinical use, the AI system automatically analyses all screening examinations and can present the results integrated into the PACS (picture archiving and communication system) user interface.

### Evaluation of the AI system

**Fig. 2:**
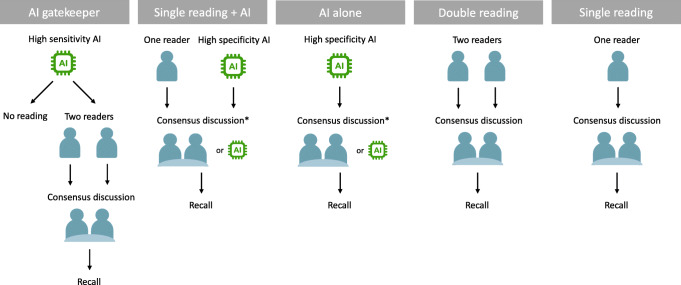
Chart of different ways of implementing AI in DBT screening workflows and workflows with human reading of DBT without AI used for comparison. AI score threshold for a high sensitivity > 3. AI score threshold for high specificity was selected separately for single reading + AI and AI alone in order to achieve about the same number of consensus discussions as DM double reading. * In cases where no actual consensus discussion is available, a surrogate consensus discussion is used by assuming recall when the AI score is among the highest 2%

The performance of the AI system on DBT was compared to radiologist double reading and single reading DBT. To reduce the DBT screen-reading workload to the same or lower level as with DM screening, several strategies for using AI were evaluated, as illustrated in Figure 2.

A. *AI Gatekeeper*, where the AI system is used to exclude normal examinations from human reading, while the other examinations are double-read. Different AI score thresholds were evaluated. Special focus was placed on a threshold where 50% of the examinations would be excluded, giving an unchanged screening workload compared to DM screening (conservative assumption of DBT reading time twice as long as DM [7–11]).
B. *Single reading + AI*, where the AI system is used to replace the second reader, also leads to a reduction by half in the total number of readings. In order to facilitate comparison, the AI system was used as a stand-alone reader with a high specificity threshold selected to have the same number of consensus discussions as with DBT double reading.
C. *AI alone*, where screening examinations are analysed only by the AI system. The AI system analysed all the examinations, and examinations were classified as sent to consensus discussion if the AI score was above a threshold where the number of discussions is the same as with DBT double reading.

Results from actual consensus discussions prompted by DBT double reading were used when available, but since no consensus discussions had taken place for cases where only AI prompted a consensus discussion, it was necessary to use surrogate consensus discussions for some cases in the *single reader + AI* and *AI alone* workflows. Since these cases had not been flagged for discussion by any of the readers, the recall rate at those consensus discussions would likely have been lower than the total recall rates for DM and DBT screening double reading arms at 2.5% and 3.6%, respectively [1]. As an approximation, cases with an AI score among the highest 2% in the population were assumed to be recalled.

For each model, the number of human readings, detected cancers, missed cancers, and false positives were investigated and compared to single and double reading DBT, respectively. Ground truth was defined using all screening-detected cancers (DM + DBT) and interval cancers. Some interval cancers might be detectable at screening, and by including interval cancers in the ground truth, the AI system is allowed to detect cancers undetected by radiologists. The number and characteristics of missed cancers were studied. The results were also compared with the current standard screening with double-read DM.

### Cancer characteristics

The number of detected and missed cancers with *AI gatekeeper*, *single reader + AI*, and *AI alone*, respectively, were calculated for subgroups including breast density (BIRADS 4^th^ edition), histological type, histological grade (for invasive cancers), nuclear grade (for *in situ* cancers), tumour size, presence of lymph node metastases and radiographic appearance. The corresponding results from double reading DM or DBT are provided for comparison.

### Statistical analyses

The distributions of the AI scores of all examinations and cancer cases were analysed with descriptive statistics, and 95% confidence intervals for proportions were calculated with the Clopper-Pearson method. The cancer detection performance of the AI system was analysed with receiver operating characteristics (ROC) with ground truth based on DBT + DM screening results combined with interval cancers. Additional analyses were performed with ground truth defined by DBT screening results and DBT + DM screening results, respectively. Corresponding AUCs were calculated, and bootstrapping with 1000 replicas was applied to yield 95% confidence intervals. Differences in the number of discussions, recalls, and sensitivity between different workflows were tested with exact McNemar’s test using R 4.0.5 (R Foundation for Statistical Computing). All other statistical analyses were performed in MATLAB 2020a (The MathWorks).

## Results

**Fig. 3:**
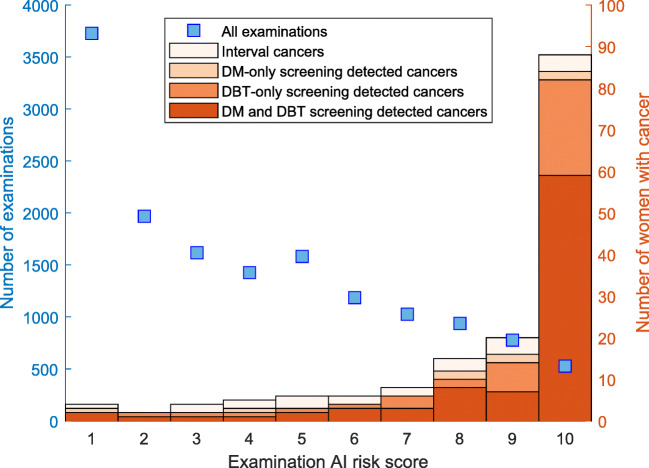
Distribution of AI scores in the whole population and cancer cases

**Fig. 4:**
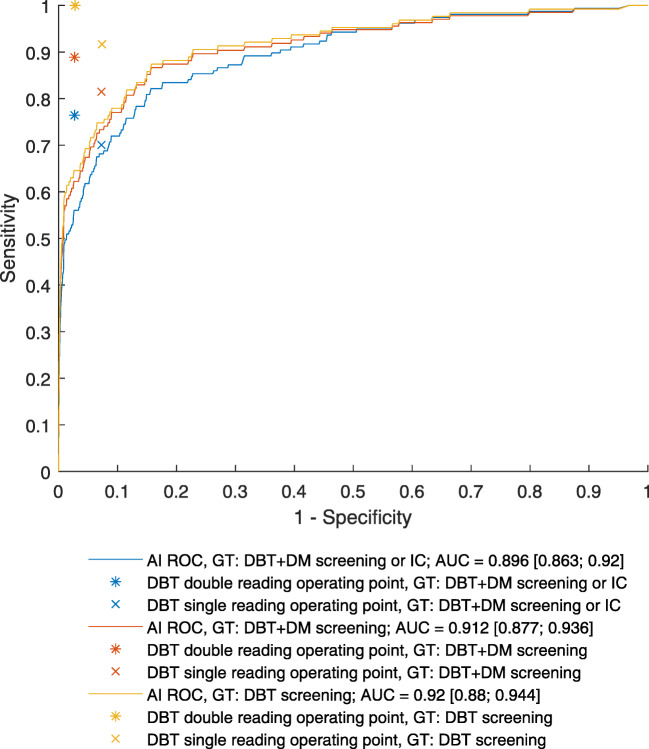
Receiver operating characteristics (ROC) analysis for cancer detection using the AI system with different definitions of ground truth (GT). Ground truth defined by DBT screening results is a direct comparison between AI and radiologists in DBT screening, but means that the radiologists by definition will be better than AI as they define the ground truth. Ground truths defined by DBT + DM screening results and DBT + DM screening results combined with interval cancers are also evaluated to theoretically allow the AI system to perform better than radiologists reading DBT. Operating points for single and double readings of DBT are provided for reference. Single-reader sensitivity is defined by using recalled after consensus discussion. Single-reader specificity is defined by using marks for discussion. IC, interval cancer

The distributions of AI scores in the whole study population and the cancer cases are presented in Figure 3. Of all cancers, 88 got an AI score of 10, while 85 of the screening-detected cancers had an AI score of 10. The ROC curves for cancer detection by the AI system with different ground truths are presented in Figure 4. The corresponding operating points for double reading and single reading DBT are shown for comparison. While the operating points for double reading are clearly higher than the corresponding ROC curves, the operating points for single reading are very close to the ROC curve. The AUC for AI system cancer detection was 0.92 with a 95% CI [0.88; 0.94] when DBT screening was used as the ground truth.

For the *AI gatekeeper* approach, an exclusion threshold of 3.0 can be considered suitable, as this would result in 7312 (49.5%) examinations being excluded from human reading (Table 1). The excluded examinations included nine cancers (6%), seven of which were screening detected, which would be missed with the *AI gatekeeper* approach. The number of false-positive recalls would be reduced by 111 (27%).

**Table 1:** Cumulative number of examinations, cancers, and false positives depending on DBT AI score threshold

AI scores	Number of examinations			Number of cancers*			Number of screening-detected cancers			Number of false-positive recalls		
≤ 1	3727	25%	[0.25; 0.26]	3	2%	[0.00; 0.05]	3	2%	[0.00; 0.06]	52	13%	[0.10; 0.17]
≤ 2	5695	39%	[0.38; 0.39]	5	3%	[0.01; 0.07]	5	4%	[0.01; 0.08]	81	20%	[0.16; 0.24]
≤ 3	7312	49%	[0.49; 0.50]	9	6%	[0.03; 0.11]	7	5%	[0.02; 0.10]	111	27%	[0.23; 0.32]
≤ 4	8738	59%	[0.58; 0.60]	14	9%	[0.05; 0.15]	10	7%	[0.04; 0.13]	146	36%	[0.31; 0.41]
≤ 5	10,320	70%	[0.69; 0.71]	20	13%	[0.08; 0.19]	13	10%	[0.05; 0.16]	194	48%	[0.43; 0.53]
≤ 6	11,505	78%	[0.77; 0.79]	26	17%	[0.11; 0.23]	17	13%	[0.08; 0.19]	229	57%	[0.52; 0.62]
≤ 7	12,531	85%	[0.84; 0.85]	34	22%	[0.15; 0.29]	23	17%	[0.11; 0.24]	272	67%	[0.63; 0.72]
≤ 8	13,468	91%	[0.91; 0.92]	49	31%	[0.24; 0.39]	35	26%	[0.19; 0.34]	313	77%	[0.73; 0.81]
≤ 9	14,245	96%	[0.96; 0.97]	69	44%	[0.36; 0.52]	51	38%	[0.30; 0.47]	356	88%	[0.85; 0.91]
Total	14,772	100%	[1.00; 1.00]	157	100%	[0.98; 1.00]	135	100%	[0.97; 1.00]	404	100%	[0.99; 1.00]

* Includes all screening-detected cancers and interval cancers

**Fig. 5:**
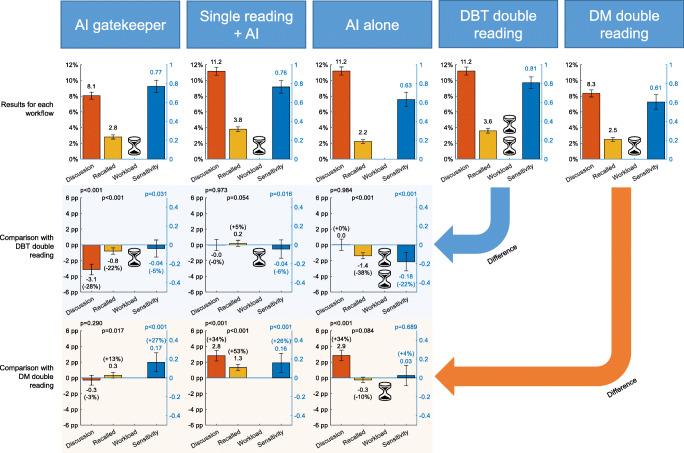
Proportion of examinations sent to consensus discussion or recalled, relative screen reading workload, and cancer detection sensitivity (ground truth defined by DM + DBT screening-detected cancers and interval cancers) for different workflows. Comparison of AI workflows with DBT double reading and DM double reading by subtraction. Discussion and recalls are on the left scale (black), while sensitivity is on the right scale (blue). Whiskers show the 95% confidence intervals calculated by normal approximation. The hourglass symbol illustrates the screen reading workload, where one hourglass equals the workload with the current standard method DM double reading. The reading time of DBT is assumed to be about double that of DM double reading. Differences in the proportion of examinations sent to discussion and recalled are given as percentage points (pp), with percentage changes in brackets. *p* values calculated with exact McNemar’s test. All calculations were performed with high precision and rounding was applied only on presented values, which may cause some small variations from expected values in the comparisons

The number of necessary screening procedures, such as readings, consensus discussions, and recalled women, for different workflows are shown in Table 2, together with the resulting number of detected cancers, missed cancers, and false-positive recalls. The proportion of cases sent to discussion, being recalled, screening workload, and sensitivity with the different workflows are illustrated in Figure 5, including comparisons with DM and DBT double reading.

**Table 2:** DBT screening procedures and number of detected cancers, depending on workflow

				All cancers			Screening-detected cancers	
	Number of readings	Number of discussions ^f^	Recalled women ^g^	Detected cancers	Missed cancers	False-positive recalls	Detected cancers	Missed cancers
AI gatekeeper ^a^	14,920	1190	415	121	36	293	121	14
Single reading + AI ^b^	14,772	1651	561	120	37	440	120	15
AI alone ^c^	0	1655	329	99	58	229	99	36
Single reading ^d^	14,772	1183	431	115	42	315	115	20
Double reading	29,544	1653	532	127	30	404	127	8
Double reading DM ^e^	29,544	1284	367	95	62	272	95	40

^a^Examinations with AI score ≤ 3 were excluded, while the other examinations were double-read

^b^Cases were sent to discussion either if marked for discussion by the first reader (flagged “Discussion” or radiologist score ≥ 3) or if the AI score is 8.74 or more. Recall decision according to actual consensus discussion if available, otherwise (424 cases) a surrogate was used recalling the 2% with the highest AI score (score threshold 9.45 resulting in 120 cases, no cancers)

^c^Cases were sent to discussion if AI score is 7.57 or more. Recall decision according to actual consensus discussion if available, otherwise (1170 cases) a surrogate was used recalling the 2% with the highest AI score (score threshold 9.45 resulting in 120 cases, no cancers)

^d^Cases were sent to discussion if marked for discussion by the first reader (flagged “Discussion” or radiologist score ≥ 3). Recall if the actual consensus discussion decided to recall

^e^Included for reference

^f^Corresponds to recall before consensus

^g^Corresponds to recall after consensus

The *AI gatekeeper* approach, focusing the radiologist reading time on the high-risk cases by double reading cases with AI scores of 4–10, would require 14,920 DBT readings and detect 121 cancers (Table 2, Fig. 5), which is 95% [0.90; 0.98] of the DBT double reading screening-detected cancers, which is a small but still significant difference (*p* = 0.031). Compared to DM screening-detected cancers, 27% more cancers would be detected, which is a significant difference (*p* < 0.001). The number of false-positive recalls with this approach is only 8% higher than with DM double reading and 27% lower than with full DBT double reading. The positive predictive value is 0.29 (121/415).

*Single reading + AI,* with an AI score threshold for consensus discussion set to obtain the same number of discussions as DBT double reading, would result in the detection of 120 cancers (Table 2, Fig. 5). That means 94% [0.89; 0.98] of the DBT double reading detected cancers, which is a minor but significant difference (*p* = 0.016). Compared to DM screening, 26% more cancers would be detected, which is significantly more (*p *< 0.001). The proportion of false-positive recalls is increased by 62%. The positive predictive value is 0.21 (120/561).

*AI alone,* with an AI score threshold selected to achieve the same number of consensus discussions as DBT double reading (Table 2, Fig. 5), would detect significantly less cancers than DBT double reading (22%, *p* < 0.001) but about the same number of cancers as DM screening with double reading (*p* = 0.689), while the number of false-positive recalls would be substantially reduced.

**Fig. 6:**
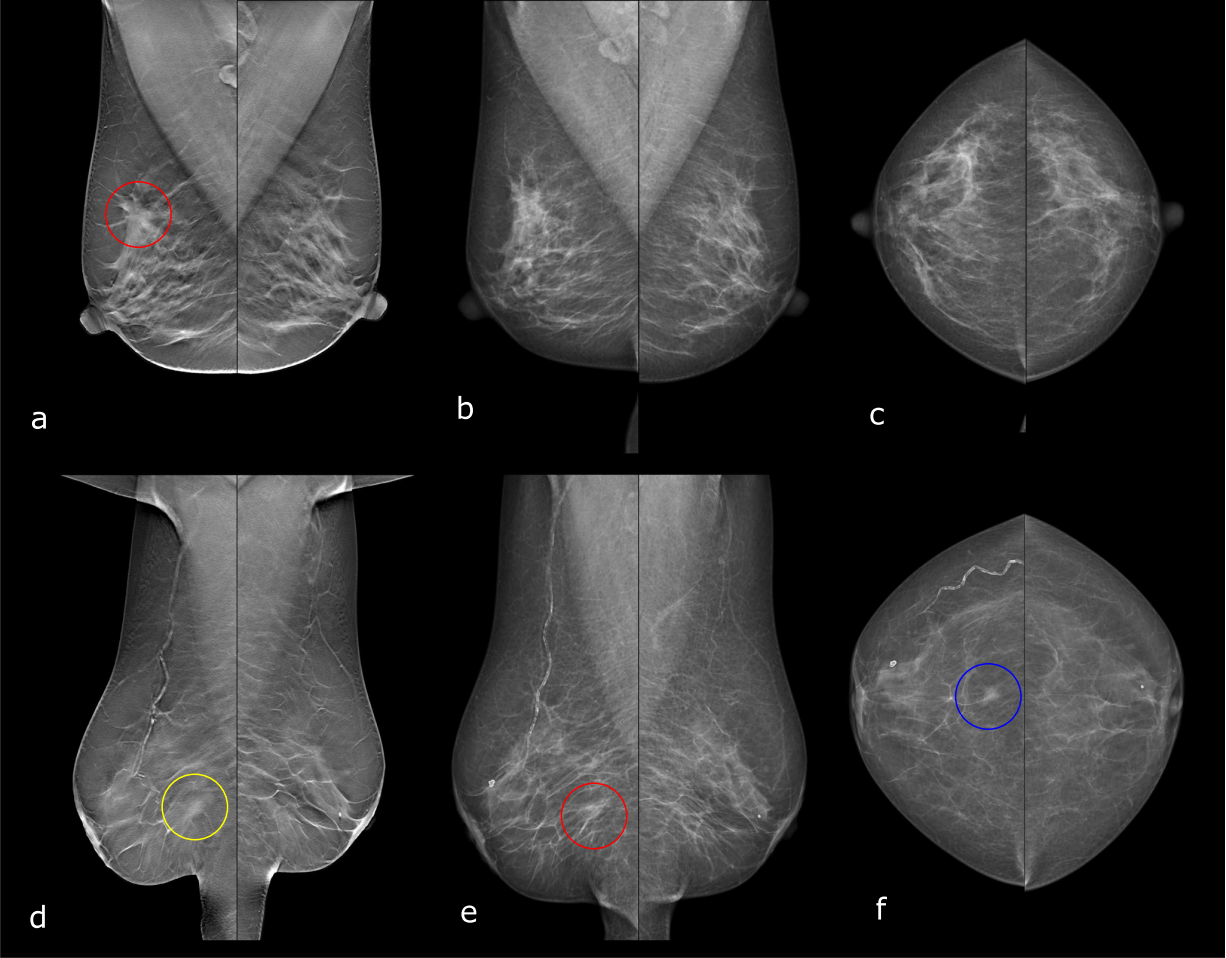
Examples of detected and missed cancers when using DBT and AI. **a–c** Example of cancer detected with AI on DBT and with DBT double reading, but not with DM double reading. DBT MLO (**a**), with the cancer identified by the AI system (red circle). DM MLO (**b**) and DM CC (**c**). The AI system gave a score of 10. The cancer was a 26 mm invasive lobular carcinoma. **d**–**f** Example of cancer missed with AI on DBT, as well as DM double reading, but detected with DBT double reading. DBT MLO (**d**), with the cancer lesion (yellow circle) not identified as suspicious of cancer by the AI system. DM MLO (**e**) and DM CC (**f**), where the readers did not detect the subtle lesion, probably due to partly overlapping tissue in the MLO projection (red circle) but clearly visible in the CC projection (blue circle). The AI system gave an examination score of 2 (yellow circle), meaning that this examination would be discarded without human reading with the AI gatekeeper workflow. The cancer was a 10 mm tubular cancer

Figure 6 shows an example of a cancer case that was detected with DBT and AI but not with DM double reading, and a cancer case that was detected with DBT double reading but missed with DBT and AI. The characteristics of all cancer cases detected using different methods are presented in Table 3. The numbers are small, but the cancers detected with *AI gatekeeper* and with *single reader + AI* exhibit no apparent differences from the DBT double-reading detected cancers, apart from a slightly lower proportion of detected *in situ* cancers. While DBT screening read by *AI alone* detects about the same number of cancers as DM double reading, a larger proportion are invasive cancers.

**Table 3:** Examination AI risk score and detection for different cancer types and characteristics

Total number		Median score	AI gatekeeper *		Single reader + AI *		AI alone *		Double reading DBT *		Double reading DM *	
All	157	9.50 (IQR = 2.44)	122	78% [0.70; 0.84]	120	76% [0.69; 0.83]	97	62% [0.54; 0.69]	127	81% [0.74; 0.87]	95	61% [0.52; 0.68]
Invasive	137	9.63 (IQR = 2.09)	110	80% [0.73; 0.87]	108	79% [0.71; 0.85]	91	66% [0.58; 0.74]	113	82% [0.75; 0.88]	81	59% [0.50; 0.67]
*In situ*	20	7.72 (IQR = 2.47)	12	60% [0.36; 0.81]	12	60% [0.36; 0.81]	6	30% [0.12; 0.54]	14	70% [0.46; 0.88]	14	70% [0.46; 0.88]
BIRADS 4th Ed breast density category (per woman)												
1	11	9.63 (IQR = 5.20)	8	73% [0.39; 0.94]	9	82% [0.48; 0.98]	7	64% [0.31; 0.89]	10	91% [0.59; 1.00]	6	55% [0.23; 0.83]
2	51	9.24 (IQR = 2.43)	37	73% [0.58; 0.84]	38	75% [0.60; 0.86]	29	57% [0.42; 0.71]	40	78% [0.65; 0.89]	29	57% [0.42; 0.71]
3	73	9.49 (IQR = 1.99)	58	79% [0.68; 0.88]	54	74% [0.62; 0.84]	47	64% [0.52; 0.75]	57	78% [0.67; 0.87]	49	67% [0.55; 0.78]
4	22	9.62 (IQR = 4.41)	19	86% [0.65; 0.97]	19	86% [0.65; 0.97]	14	64% [0.41; 0.83]	20	91% [0.71; 0.99]	11	50% [0.28; 0.72]
Histological type												
Invasive ductal cancer	93	9.60 (IQR = 2.41)	71	76% [0.66; 0.85]	70	75% [0.65; 0.84]	59	63% [0.53; 0.73]	72	77% [0.68; 0.85]	54	58% [0.47; 0.68]
Invasive lobular cancer	26	9.62 (IQR = 1.91)	23	88% [0.70; 0.98]	20	77% [0.56; 0.91]	18	69% [0.48; 0.86]	23	88% [0.70; 0.98]	14	54% [0.33; 0.73]
Tubular cancer	17	9.74 (IQR = 1.77)	15	88% [0.64; 0.99]	17	100% [0.80; 1.00]	13	76% [0.50; 0.93]	17	100% [0.80; 1.00]	12	71% [0.44; 0.90]
Ductal carcinoma *in situ*	20	7.72 (IQR = 2.47)	12	60% [0.36; 0.81]	12	60% [0.36; 0.81]	6	30% [0.12; 0.54]	14	70% [0.46; 0.88]	14	70% [0.46; 0.88]
Other	1	9.80 (IQR = 0.00)	1	100% [0.03; 1.00]	1	100% [0.03; 1.00]	1	100% [0.03; 1.00]	1	100% [0.03; 1.00]	1	100% [0.03; 1.00]
Histological grade, invasive cancers												
1	46	9.73 (IQR = 1.16)	41	89% [0.76; 0.96]	44	96% [0.85; 0.99]	35	76% [0.61; 0.87]	43	93% [0.82; 0.99]	31	67% [0.52; 0.80]
2	62	9.44 (IQR = 2.68)	48	77% [0.65; 0.87]	46	74% [0.62; 0.84]	36	58% [0.45; 0.70]	49	79% [0.67; 0.88]	34	55% [0.42; 0.68]
3	22	9.72 (IQR = 1.47)	17	77% [0.55; 0.92]	15	68% [0.45; 0.86]	16	73% [0.50; 0.89]	17	77% [0.55; 0.92]	13	59% [0.36; 0.79]
Nuclear grade, *in situ* cancers												
1	2	7.22 (IQR = 1.32)	1	50% [0.01; 0.99]	1	50% [0.01; 0.99]	0	0% [0.00; 0.84]	1	50% [0.01; 0.99]	1	50% [0.01; 0.99]
2	7	8.32 (IQR = 4.60)	3	43% [0.10; 0.82]	4	57% [0.18; 0.90]	2	29% [0.04; 0.71]	4	57% [0.18; 0.90]	4	57% [0.18; 0.90]
3	10	7.53 (IQR = 2.74)	8	80% [0.44; 0.97]	7	70% [0.35; 0.93]	4	40% [0.12; 0.74]	9	90% [0.55; 1.00]	9	90% [0.55; 1.00]
Size, pathology size												
< = 10	51	9.57 (IQR = 2.86)	40	78% [0.65; 0.89]	42	82% [0.69; 0.92]	31	61% [0.46; 0.74]	45	88% [0.76; 0.96]	33	65% [0.50; 0.78]
11–15	51	9.60 (IQR = 2.68)	41	80% [0.67; 0.90]	39	76% [0.63; 0.87]	32	63% [0.48; 0.76]	42	82% [0.69; 0.92]	29	57% [0.42; 0.71]
16–20	23	9.63 (IQR = 2.60)	19	83% [0.61; 0.95]	19	83% [0.61; 0.95]	16	70% [0.47; 0.87]	18	78% [0.56; 0.93]	15	65% [0.43; 0.84]
> 20	32	9.15 (IQR = 1.79)	24	75% [0.57; 0.89]	22	69% [0.50; 0.84]	20	63% [0.44; 0.79]	24	75% [0.57; 0.89]	22	69% [0.50; 0.84]
Missing	6	8.78 (IQR = 5.67)	2	33% [0.04; 0.78]	2	33% [0.04; 0.78]	2	33% [0.04; 0.78]	2	33% [0.04; 0.78]	1	17% [0.00; 0.64]
Axillary lymph node status												
Negative	96	9.64 (IQR = 1.99)	85	89% [0.80; 0.94]	85	89% [0.80; 0.94]	68	71% [0.61; 0.80]	89	93% [0.86; 0.97]	63	66% [0.55; 0.75]
Positive	28	9.80 (IQR = 1.08)	27	96% [0.82; 1.00]	26	93% [0.76; 0.99]	24	86% [0.67; 0.96]	27	96% [0.82; 1.00]	21	75% [0.55; 0.89]
Missing	13	7.56 (IQR = 2.49)	10	77% [0.46; 0.95]	9	69% [0.39; 0.91]	5	38% [0.14; 0.68]	11	85% [0.55; 0.98]	11	85% [0.55; 0.98]
Radiographic appearance												
Spiculated mass	90	9.80 (IQR = 1.14)	84	93% [0.86; 0.98]	84	93% [0.86; 0.98]	70	78% [0.68; 0.86]	87	97% [0.91; 0.99]	62	69% [0.58; 0.78]
Circumscribed mass	18	8.90 (IQR = 2.31)	16	89% [0.65; 0.99]	16	89% [0.65; 0.99]	12	67% [0.41; 0.87]	18	100% [0.81; 1.00]	11	61% [0.36; 0.83]
Calcifications	22	8.27 (IQR = 2.18)	16	73% [0.50; 0.89]	14	64% [0.41; 0.83]	10	45% [0.24; 0.68]	17	77% [0.55; 0.92]	20	91% [0.71; 0.99]
Distortion	4	8.85 (IQR = 1.64)	4	100% [0.40; 1.00]	4	100% [0.40; 1.00]	3	75% [0.19; 0.99]	4	100% [0.40; 1.00]	1	25% [0.01; 0.81]
Invisible	1	9.80 (IQR = 0.00)	1	100% [0.03; 1.00]	1	100% [0.03; 1.00]	1	100% [0.03; 1.00]	1	100% [0.03; 1.00]	1	100% [0.03; 1.00]

AI results based on analysis of DBT examinations. DM and DBT screening detection based on double reading with consensus

*Values in brackets are 95% confidence intervals calculated with the Clopper–Pearson method. *IQR*, interquartile range

## Discussion

We retrospectively evaluated an AI system for cancer detection in DBT examinations and studied different ways of implementing this system in a screening workflow to reduce the DBT reading workload, with the aim of exploring the field and building a basis for future prospective studies. An *AI gatekeeper* approach, where the AI system excludes low-risk cases and instead focuses radiologists’ time on double-reading high-risk cases, was shown to detect almost all the cancers detected with DBT double reading while reducing the number of false-positive recalls. With a *single reader + AI*, where AI replaces the second reader, slightly fewer cancers would be detected, and under the assumptions used in this retrospective study, an increase in false-positive recalls was found. The *AI-alone* approach to DBT could reach the level of sensitivity of DM screening without human readers.

### AI as a stand-alone reader

As a stand-alone reader, the AI system performs on a par with single reading but is still inferior to double reading of DBT. In the MBTST, DBT double reading detected significantly more cancers than DM double reading [1]. However, this study shows only a small difference in AI AUC, with 0.90 at DBT compared to 0.88 at DM in the same population [30]. This might be a sign that AI for DBT still cannot utilise all the additional information in DBT. The availability of DBT training data is still limited, but in the future, more training data might improve performance, and new applications may emerge.

### Approaches for workload reduction and clinical implications

Both the *AI gatekeeper* and the *single reader + AI* approaches could almost halve the number of DBT readings, which, if DBT reading time was assumed to be about 75% longer than DM reading time, would lead to a reduction in total reading workload, even compared to baseline DM screening. With the *AI gatekeeper*, the number of false-positive recalls would be almost the same as with DM screening. This could increase the cost-effectiveness of DBT screening enough to enable the introduction of DBT in population-based screening programmes, and more cancers could be detected without large increases in screening expenses. The recall rate from *single reader + AI* would probably be lower in a prospective situation where the reader used AI interactively and real consensus discussions were held to select cases of recall, and would probably not differ considerably from that of the *AI gatekeeper*. However, the transition to DBT screening would require further education of breast radiologists followed by a learning phase. The reading workload could be further reduced if *AI alone* were used on one-view DBT and still performed on a par with two-view DM double reading, with a slightly lower radiation dose, and *AI alone* could be an option in cases where reading resources are very limited. No clear differences in detection among cancers with different characteristics were shown in this study, but the possibility that such differences might appear if a larger population were to be studied cannot be excluded.

Changing the workflow might affect the readers, which could limit generalisability to the prospective situation. The composition of the screening population seen by the radiologists would be affected in an *AI gatekeeper* workflow. Since the workflow would otherwise be unchanged with double reading and consensus discussions, the effects on reading performance ought to be limited. In *single reader + AI*, knowledge of being the only reader could increase discussion or recall rates. The surrogate consensus discussions are based only on AI results, while real consensus discussions combine AI findings, radiologist interpretation, and screening history and would probably result in another selection of cases for recall.

Previous studies have proposed the use of AI as a decision support tool for a single-reading radiologist [23, 24, 31], which might increase single-reader cancer detection but comes with the risk of increasing false-positive recalls for areas identified by AI. Heavily relying on AI findings might reduce sensitivity for cancers not detected by AI.

Implementing AI in the workflow in a way that replaces a human reader can be a big step both legally and psychologically. An approach with a *single reader + AI* would probably more easily gain acceptance and be easier to implement, since all examinations would still be read by a human. Of women taking part in breast cancer screening, 59% reported they would trust a computer-only reading, while 84% would trust AI combined with a human reader [32]. A study of mammography AI preferences among primary care providers reported an equal inclination to recommend both approaches [33]. However, with AI successively becoming more common in various applications, AI will probably grow to become more accepted and trusted.

Considering the different aspects of the different screening methods, we believe that *single reader + AI* could be the easiest way to implement AI in DBT screening, as this would avoid many of the legal and psychological obstacles of implementing AI as a sole reader. This advantage probably outweighs the advantage of the slightly better performance seen with the *AI gatekeeper*.

### Comparison with previous studies

This study confirms a previous study that showed a 25% increase in sensitivity compared to DM double reading, with AI-triaged two-view DBT screening excluding about 70% of the examinations from human reading [19]. However, the comparison with our study is complicated by differences in the study design and characteristics of the screening programme and population. The AUC in our study is somewhat lower than in two previous studies of stand-alone AI on a DBT screening material [18, 20] and slightly higher than in one previous study [21].

### Limitations

This study has some limitations, including being a single-centre study with images acquired with a single vendor DM/DBT unit and being analysed with a single AI system. The reading time was not measured, but had to be estimated from the literature; therefore, precise effects on the total reading workload cannot be concluded. One-view DBT was used, while screening with two-view DBT could detect more cancers and has also been more thoroughly studied in a number of studies [2, 3, 7]. The use of AI with two-view DBT might provide different results and should also be studied. While including interval cancers in the ground truth allows the AI to find undetected cancers, including cancers detected at the following screening would further increase the possibility of undetected cancers being found. Furthermore, this study is retrospective, meaning that it cannot analyse how the use of AI affects readers. The use of AI as decision support has not been studied. The consensus discussions did not include AI results, which could potentially have affected decisions. The surrogate consensus discussions were based only on AI scores without any assessment by radiologists.

## Conclusions

AI can reduce the reading workload of DBT screening by either excluding low-risk cases from readings or replacing the second reader. This could enable us to gain most of the benefits of DBT screening without increasing the total reading workload and thus advance the implementation of DBT in screening. Considering the legal and psychological obstacles to having examinations not read by a human, the replacement of the second reader with AI might be the most feasible strategy. Alternatively, AI can replace both human readers in DBT screening, with the same sensitivity as DM double reading and fewer false-positive recalls. Prospective studies are needed to obtain more realistic consensus discussion results and investigate how AI affects the behaviour of readers.

## Abbreviations

AI: Artificial intelligence
CC: Craniocaudal
DBT: Digital breast tomosynthesis
DM: Digital mammography
MBTST: Malmö breast tomosynthesis screening trial
MLO: Mediolateral oblique

## Declarations

### Guarantor

The scientific guarantor of this publication is Sophia Zackrisson.

### Statistics and biometry

One of the authors has significant statistical expertise.

### Methodology

• retrospective

• experimental

• performed at one institution
